# To the Editors of the Medical and Physical Journal

**Published:** 1803-01-01

**Authors:** John Walker

**Affiliations:** No. 54, Lombard Street


### *To the Editors of the Medical and Physical Journal.*

GENTLEMEN,

IN the last volume of a very popular work, (Public Characters, 1802, 1803,) I read, in the otherwise well-written Life of JENNER, a mistake which feels very unpleasant to me; inasmuch as it goes to charge Government (the naval and military department of it) with a meanness or littleness, which is not correctly stated. It is there said, that the remuneration granted to the two medical men who introduced vaccination into the British garrisons, &c. up the Mediterranean, and one of whom accompanied the late expedition to Egypt, was fifty pounds. The fact is, the War Office have given us two hundred pounds; and in Evan Nepean's letter to me, 5 Feb. 1802, he says, "I am commanded by their Lordships to acquaint you, that they have been pleased to order the Navy Board to repay you the amount of the disbursements which you and Dr. Marshall have made, and to present you with the sum of one hundred pounds in consideration of such services. I am, Sir, &c."

The hundred pounds have been paid, but government yet continues Debtor the disbursements.

Now, there being few medical men who will not read, with great interest, whatever relates to the author of that discovery, whereby England may be said to have furnished to the world new means of health, and to have opened out to medical science a new field for speculation, in which other pleasant fruits may hereafter be gathered than those which we now derive from the dairy, though we may still continue utterly at a loss as to the manner of their growth; and your Journal also being in general circulation, will ye be kind enough to insert the above in it? Medical men may be rather gratified to find, that Government has not treated their profession so scurvily as they might have otherwise supposed.

I ought to mention also, that besides the disbursements not yet refunded, there occurred in my Memorial to the two Offices, the following, perhaps, drawback from their remuneration. "The Governor and a few others at the Rock (Gibraltar) chose to fee us for attendance in their respective families. The master of an American vessel had his ship's company inoculated, and also fee'd us. At Minorca a very liberal fee, presented in the most flattering and and affectionate way, for a medical attendance, at the government house, was about equal to what was had from all the other inhabitants. At Malta they propose to send each of us a piece of plate. In Egypt I received a present from one of the Beys, for visiting Turkish officers at his house, and soldiers in the hospitals at Rosetta; he apologizing for its smallness, by saying, that his resources had been diminished by the presence of the French."

"The intention, (I hope it will be considered a candid one) of mentioning these circumstances, is, that if government shall think it right to reward our services (for I think by the laws of our country the profession of the physician is on the footing, perhaps flattering to him, that he cannot make a demand) it may know, that, during our absence from England, *every* hour has not been employed by us in its immediate service.

"Our fees I have not kept an account of; but they did not altogether amount to one hundred pounds."*

*No.* 54, *Lombard Street,* I am, &c.

*December* 12, 1802. JOHN WALKER.

* From this account the liberal remuneration of the Court of Naples, and all the fees obtained in Sicily and Italy, are excluded, as of subsequent date to the subject of the Memorial.

